# Associations of 26 Circulating Inflammatory and Renal Biomarkers with Near-Infrared Spectroscopy and Long-term Cardiovascular Outcome in Patients Undergoing Coronary Angiography (ATHEROREMO-NIRS Substudy)

**DOI:** 10.1007/s11883-018-0752-8

**Published:** 2018-09-14

**Authors:** Sharda S. Anroedh, K. Martijn Akkerhuis, Rohit M. Oemrawsingh, Hector M. Garcia-Garcia, Milos Brankovic, Evelyn Regar, Robert-Jan van Geuns, Patrick W. Serruys, Joost Daemen, Nicolas M. van Mieghem, Eric Boersma, Isabella Kardys

**Affiliations:** 1 Department of Cardiology, Erasmus MC, Room Na-316, P.O. Box 2040, 3000 CA Rotterdam, The Netherlands; 2 Cardiovascular Research School COEUR, Erasmus MC, Rotterdam, The Netherlands; 3 Department of Cardiology, Erasmus MC, Room Rg-4, P.O. Box 2040, 3000 CA Rotterdam, The Netherlands; 4 Department of Cardiology, Erasmus MC, Room Rg-6, P.O. Box 2040, 3000 CA Rotterdam, The Netherlands; 5 Section of Interventional Cardiology, MedStar Washington Hospital Center, Washington, DC USA; 6 Division of Cardiovascular Surgery, University Hospital Zurich, Zurich, Switzerland; 7 Department of Cardiology, Radboud UMC, Geert Grooteplein Zuid 10, 6525 GA Nijmegen, The Netherlands; 8 Cardiovascular Science Division, National Heart & Lung Institute, Imperial College London, London, SW7 2AZ UK; 9 Department of Cardiology, Erasmus MC, Room Na-319, P.O. Box 2040, 3000 CA Rotterdam, The Netherlands

**Keywords:** Clinical research, Cardiovascular disease, Atherosclerosis, Biomarkers, Intracoronary imaging, Long-term follow-up

## Abstract

**Purpose of Review:**

The purpose of this study was to investigate the association of 26 inflammatory biomarkers (acute phase proteins, cytokines, chemokines) and renal markers with coronary lipid core burden index (LCBI) assessed by near-infrared spectroscopy (NIRS) imaging, as well as the association of these biomarkers with long-term cardiovascular outcome.

**Recent Findings:**

NIRS-derived LCBI has recently been shown to be an independent predictor of major adverse cardiac events (MACE). However, studies on the association between circulating biomarkers and NIRS-derived characteristics have not yet been performed.

**Summary:**

Between 2008 and 2011, 581 patients underwent diagnostic coronary angiography or percutaneous coronary intervention for stable angina pectoris or acute coronary syndrome (ACS). NIRS of a non-culprit vessel was performed in a subset of 203 patients. In multivariable analyses, TNF-α tended to be associated with higher LCBI (beta 0.088 ln (pg/ml) increase per unit LCBI; 95% CI 0.000–0.177, *p* = 0.05) after adjustment for clinical characteristics. However, this association did not persist after Bonferroni correction (statistical threshold 0.0019). Major adverse cardiac events (MACE) were registered in 581 patients during a median follow-up time of 4.7 years (IQR: [4.2–5.6] years). After adjustment for clinical characteristics and Bonferroni correction, IL-8 (HR 1.60; 95% CI [1.18–2.17] per ln (pg/ml), *p* = 0.002) was borderline associated with MACE and significantly associated with all-cause mortality or ACS (HR 1.75; 95% CI [1.24–2.48] per ln (pg/ml), *p* = 0.0015). In conclusion, we found that IL-8 was independently associated with clinical outcome, but altogether, the multiplex panel we investigated here did not render a useful blood biomarker of high LCBI.

**Electronic supplementary material:**

The online version of this article (10.1007/s11883-018-0752-8) contains supplementary material, which is available to authorized users.

## Introduction

Vulnerable plaque, defined as a plaque that is sensitive to rupture [1], is characterized by a large lipid core, thin fibrous cap, and active inflammation [2]. Pathology studies have shown that approximately 60% of acute coronary syndromes (ACS) are caused by ruptures of such vulnerable plaques [3]. Near-infrared spectroscopy (NIRS) is a novel catheter-based imaging technique based on diffuse reflectance spectroscopy [4]. This technique is capable of characterizing the chemical components of the atherosclerotic plaque and is consequently able to identify lipid core [5•]. Lipid core plaques (LCP) have been shown to be more vulnerable to rupture than non-LCP [6••, 7]. A strong association has been demonstrated between LCP, as detected by NIRS, and cardiovascular events [6••].

An alternative, non-invasive way to detect LCP could aid in risk stratification. Blood biomarkers may carry potential to detect vulnerable plaques in an early stage and in a non-invasive manner. Among others, biomarkers of inflammation (such as acute phase proteins, cytokines, and chemokines) and renal markers have strongly been implicated in the atherosclerotic process and in the occurrence of coronary events [7–12]. Currently, to the best of our knowledge, there are no data available on the associations between circulating biomarkers and NIRS measurements. Such an investigation could lead to further pathophysiological insights concerning plaque vulnerability, and could help bridge the gap between known biological pathways and clinical imaging findings.

Therefore, the purpose of this study was to investigate the association of 26 circulating inflammatory and renal biomarkers with coronary lipid core burden index (LCBI) as determined in vivo by NIRS imaging in patients undergoing coronary angiography. Furthermore, the long-term prognostic value of these biomarkers for the occurrence of major adverse cardiac events (MACE) was evaluated.

## Methods

### Study Population and Design

**Fig. 1:**
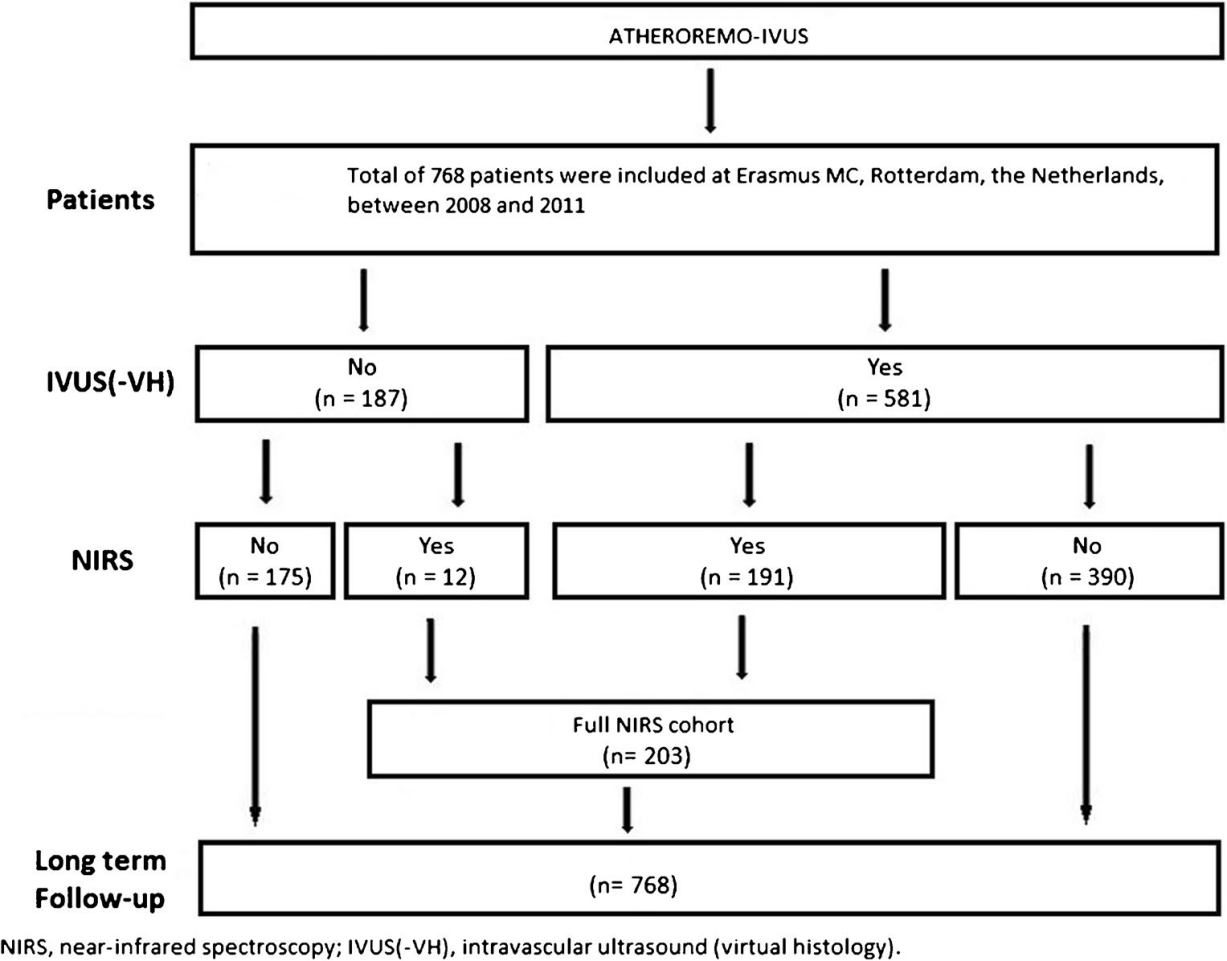
Patient inclusion. IVUS of a non-culprit artery was performed in 581 patients and blood samples were available in 570 patients. NIRS of a non-culprit artery was performed in 203 patients and blood samples were available in 190 patients

The design of The European Collaborative Project on Inflammation and Vascular Wall Remodeling in Atherosclerosis–Intravascular Ultrasound (ATHEROREMO-IVUS), and its substudy the European Collaborative Project on Inflammation and Vascular Wall Remodeling in Atherosclerosis–Near-Infrared Spectroscopy (ATHEROREMO-NIRS), has been described elsewhere [6••, 13•]. In brief, from 2008 until 2011, 768 patients with an indication for diagnostic coronary angiography (DCO) or percutaneous coronary intervention (PCI) due to stable angina pectoris (SAP) or ACS were included in a biomarker study in Erasmus MC, Rotterdam, the Netherlands. In 581 of these patients, IVUS of a non-culprit vessel was performed (Fig. 1). Among these patients, NIRS of the same segment was performed in a subset of 191 patients. In 12 additional patients, only NIRS, not IVUS, was performed, rendering a total of 203 patients in whom NIRS measurements were available.

Both studies were approved by the medical ethics committee of the Erasmus MC and were performed in accordance with the criteria described in the declaration of Helsinki. Written informed consent was obtained from all included patients.

### Biomarker Measurements

Blood samples were drawn from the arterial sheath prior to the diagnostic coronary angiography or PCI procedure and were stored at the clinical laboratory of Erasmus MC at a temperature of − 80 °C within 2 h after blood collection. C-reactive protein (CRP) was measured at Erasmus MC in serum using an immunoturbidimetric high-sensitivity assay (Roche Diagnostics Ltd., Rotkreuz, Switzerland) on the Cobas 8000 modular analyzer platform (Roche Diagnostics Ltd., Rotkreuz, Switzerland). Frozen EDTA-plasma samples were transported under controlled conditions (at a temperature of − 80 °C) to Myriad RBM, Austin, Texas, USA, where the concentrations of cytokines, chemokines, acute-phase proteins, and renal biomarkers were determined using a validated multiplex assay (Custom Human Map, Myriad RBM, Austin, Texas, USA). CRP, ferritin, haptoglobin, plasminogen activator inhibitor 1 (PAI 1), fibrinogen, macrophage inflammatory protein-1 alpha (MIP-1 α), macrophage inflammatory protein-1 beta (MIP-1 β), monocyte chemotactic protein 1 (MCP-1), regulated upon activation normal T cell expressed and secreted (T cell-specific RANTES), tumor necrosis factor receptor 2(TNF R2), interleukin-6 (IL-6), interleukin-8 (IL-8), creatinine, cystatin C, adiponectin, and myoglobin were determined in 570 patients from the ATHEROREMO-IVUS population (*n* = 581) and 190 patients of the ATHEROREMO-NIRS population (*n* = 203). Alpha-1-antitrypsin (AAT), alpha-2-macroglobulin (A2Macro), complement C 3 (C3), tumor necrosis factor alpha (TNF-α), tumor necrosis factor beta (TNF-β), interferon γ (INF-γ), Interleukin-10 (IL-10), interleukin-18 (IL-18), neutrophil gelatinase-associated lipocalin (NGAL), and beta-2-microglobulin (B2M) were determined in random subsets of 473 and 156 patients, respectively*.* This difference in numbers resulted from batch-wise handling of the samples in combination with an update of the composition of the multiplex assay by the manufacturer in-between two batches. The biomarker laboratories had no knowledge of clinical or intracoronary imaging data.

### Near-Infrared Spectroscopy

**Fig. 2:**
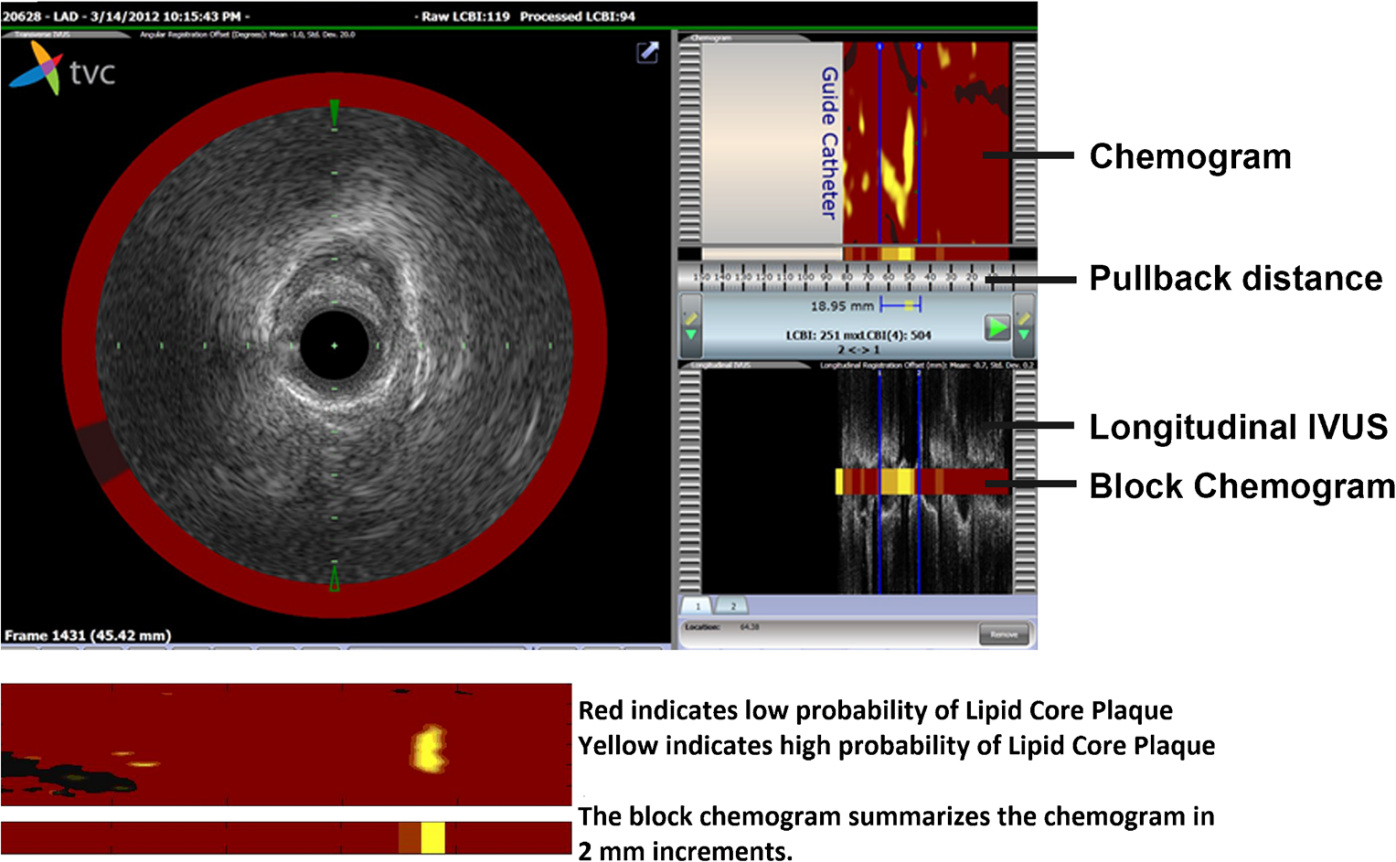
Intracoronary near-infrared spectroscopy displayed as a chemogram. The figure displays an example of coronary wall imaging by near-infrared spectroscopy. Spectral characteristics of lipid core plaques are displayed on a chemogram along the length (*x*-axis, in mm) and circumference (*y*-axis, 0 to 360°) of the scanned coronary artery. Yellow regions in the chemogram represent high probability of LCP while red regions represent those with low probability of LCP. The LCBI quantifies the amount of LCP in the entire scanned artery segment on the block chemogram, and is computed as the fraction of valid pixels that exceed an LCP probability of 0.6, multiplied with 1000

The NIRS coronary imaging system consisted of a 3.2-F rapid exchange catheter, a pullback and rotation device, and a console (InfraReDx, Burlington, Massachusetts, USA). This NIRS system was approved by the U.S. Food and Drug Administration. The NIRS image acquisition was performed in a non-culprit vessel. The order of preference for selection of the non-culprit vessels was predefined in the study protocol: (1) left anterior descending artery; (2) right coronary artery; and (3) left circumflex artery. The NIRS target segment of the non-culprit vessel was required to be at least 40 mm in length and without significant luminal narrowing (< 50% stenosis) as assessed by online angiography. Image acquisition was performed by a motorized catheter pullback at a speed of 0.5 mm/s and 240 rpm in a proximal segment of the artery, starting distal to a side branch. Immediately after a pullback, the data in the scanned coronary arterial segment were displayed in a chemogram. The probability of the presence of LCP in the scanned coronary arterial segment was calculated by means of a prediction algorithm and was displayed using colors, ranging from red (low probability of LCP) to yellow-coded plaque (high probability of LCP) [14] (Fig. 2). The *x*-axis of the chemogram represents the pullback position in millimeters and the *y*-axis the degree of rotation within the artery from 0 to 360°. The block chemogram summarizes the chemogram in 2 mm increments. The numeric value of each block in the block chemogram is the 90th percentile of all pixel values in the corresponding 2-mm chemogram segment [15]. The block chemogram is mapped to the same color scale as the chemogram, but the display is binned to four discrete colors to aid in visual interpretation (red: *p* < 0.57, orange: 0.57 < *p* ≤ 0.84, tan: 0.84 < *p* ≤ 0.98, and yellow: *p* > 0.98, with *p* being the algorithm probability that a LCP is present in that 2-mm block) [15]. The LCBI quantifies the amount of LCP in the entire scanned artery segment on the block chemogram, and is computed as the fraction of valid pixels that exceed an LCP probability of 0.6, multiplied with 1000 [15]. NIRS images were evaluated offline by an independent core research laboratory (Cardialysis BV, Rotterdam, the Netherlands) that had no knowledge of any other patient, biomarker, or outcome data.

### Follow-up and Study Endpoints

Clinical and vital status of patients were collected from medical charts, civil registries, or by written or telephone contact with the patients or relatives. Specifically, all living patients participating in the IVUS/NIRS study were systematically questioned on the occurrence of MACE and re-admission. For patients with adverse events, hospital discharge letters were obtained and treating physicians or hospitals were contacted if necessary for additional information. The primary endpoint was the occurrence of MACE, defined as the composite of all-cause mortality, nonfatal ACS, or unplanned coronary revascularization. The secondary endpoint was the composite of all-cause mortality or nonfatal ACS.

Endpoints were adjudicated by a clinical events committee that was blinded for biomarker data and IVUS/NIRS imaging characteristics. ACS was defined as the clinical diagnosis of ST segment elevation myocardial infarction (STEMI), non-STEMI, or unstable angina pectoris (UAP) in accordance with the guidelines of the European Society of Cardiology [16, 17]. Unplanned coronary revascularization was defined as unplanned PCI or unplanned coronary artery bypass grafting (CABG).

### Statistical Analysis

The distributions of continuous variables, including biomarkers and NIRS measurements, were examined for normality by visual inspection of the histogram and calculation of the skewness coefficient. Normally distributed continuous variables are presented as mean ± standard deviation (SD), while non-normally distributed continuous variables are presented as median (interquartile range [IQR]) and were logarithmically (Ln) transformed for further analyses. For reasons of uniformity, all biomarkers are presented as median (IQR). Categorical variables are presented as numbers and percentages. All analyses were performed in the full cohort and subsequently in patients with ACS and patients with SAP separately, to investigate possible heterogeneity.

We examined the association between biomarker concentrations and LCBI as assessed by NIRS using linear regression with LCBI as the independent variable and continuous biomarkers as the dependent variable. The concentrations of CRP, A2Macro, ferritin, haptoglobin, PAI-1, MIP-1α, MIP-1β, MCP-1, T cell-specific RANTES, TNF-α, TNF-β, TNFR2, INF-γ, IL-6, IL-8, IL-10, IL-18, fibrinogen, creatinine, cystatin C, NGAL, adiponectin, myoglobin, and B2M were not normally distributed and therefore Ln transformed. TNF-β and IL-6 were too low to detect in a large part of the patients and thus were not examined as continuous variables but as categorical variables (measurable vs non-measurable). The results are presented as beta coefficients (B) that indicate unit increase in (Ln-transformed) biomarker per unit increase in Ln-transformed LCBI measurement, with 95% confidence intervals (CI).

Cox proportional hazards models were used to examine the associations between biomarker concentrations and MACE, as well as the composite of all-cause mortality or nonfatal ACS. Results are presented as hazard ratios (HRs) per unit increase in (Ln-transformed) biomarker concentration or per category of biomarker concentration, with 95% CIs. Patients lost to follow-up were considered at risk until the date of last contact, at which time point they were censored. For patients with more than one event, the first was considered. To test effect modification, interaction terms were entered into the models consisting of the product of biomarker and indication for angiography (ACS or SAP).

First, all above-described analyses were performed univariably. Based on existing literature, age (continuous variable), as well as sex, hypertension, hypercholesterolemia, and diabetes mellitus (all categorical variables), were considered as potential confounders and were subsequently entered as covariates into the multivariable analyses. In the full cohort, indication for coronary angiography was also entered as a covariate.

All statistical tests were two-tailed. *p* values < 0.05 were considered statistically significant and the results are presented with 95% confidence intervals (95% CIs). Subsequently, the Bonferroni correction was applied to account for the 26 biomarkers that were investigated (and thus *p* values < 0.05/26, i.e., *p* < 0.0019, were considered statistically significant). Data were analyzed with SPSS software (SPSS 23.0 IBM Corp., Armonk, NY, USA).

## Results

### Baseline Characteristics

Baseline clinical and imaging characteristics are summarized in Table 1 and Supplemental Table 1. In ATHEROREMO-NIRS (*n* = 203), mean age was 63.4 years, and 72.9% were men. The biomarker concentrations are presented in Supplemental Table 2 and Supplemental Table 3. In the full cohort (*n* = 570), serum concentrations of MIP-1α, TNF-α, TNF-β, IL-6, and NGAL were measurable in 84%, 92%, 8%, 38%, and 97% of the patients, respectively. The remaining biomarker concentrations were measurable in ≥ 99% of the patients. In the ATHEROREMO-NIRS cohort, concentrations of TNF-β, IL-6, and NGAL were measurable in 6%, 32%, and 96% of the patients, respectively. The remaining biomarker concentrations were measurable in ≥ 99% of the patients.

**Table 1:** Baseline clinical and procedural characteristics (ATHEROREMO-NIRS cohort, *n* = 203)

	Total	ACS patients	SAP patients
	(*n* = 203)	(*n* = 95)	(*n* = 108)
Clinical characteristics			
Age, years, mean ± standard deviation	63.4 ± 10.9	62 ± 11.7	64.7 ± 10.2
Male	148(72.9)	63(66.3)	85(78.7)
Diabetes mellitus	41(20.2)	17(17.9)	24(22.2)
Hypertension	114(56.2)	51(53.7)	63(58.3)
Hypercholesterolemia	115(56.7)	43(45.3)	72(66.7)
Smoking	50(24.6)	30(31.6)	20(18.5)
Positive family history of CAD	120(59.1)	51(54.3)	69(63.9)
Previous MI	79(38.9)	34(35.8)	45(41.7)
Previous PCI	78(38.4)	27(28.4)	51(47.2)
Previous CABG	6(3.0)	2(2.1)	4(3.7)
Previous stroke	6(3.0)	4(4.2)	2(1.9)
Peripheral artery disease	11(5.4)	5(5.3)	6(5.6)
History of heart failure	9(5.9)	3(3.2)	6(5.6)
Procedural characteristics			
Indication for coronary angiography			
ACS	95(46.8)	95(100)	–
Acute MI	28(13.8)	28(29.5)	–
Unstable angina pectoris	67(33.0)	67(70.5)	–
Stable angina pectoris	108(53.2)	–	108(100)
PCI performed	179(88.2)	88(92.6)	91(84.3)
Coronary artery disease^1^			
No significant stenosis	16(7.9)	8(8.4)	8(7.4)
1-vessel disease	106(52.2)	49(51.6)	57(52.8)
2-vessel disease	58(28.6)	26(27.4)	32(29.6)
3-vessel disease	23(11.3)	12(12.6)	11(10.2)
NIRS characteristics			
Median LCBI [IQR]	43.0[15.0–90.0]	47.0[16.0–90.0]	35.0[14.0–85.5]
Imaged coronary artery			
Left anterior descending	74(36.5)	41(43.2)	33(30.6)
Left circumflex	70(34.5)	30(31.6)	40(37.0)
Right coronary artery	59(29.1)	24(25.3)	35(32.4)

Continuous variables are presented as mean ± standard deviation (SD) or median [IQR]. Categorical variables are presented in numbers (*n*) and percentages (%). ACS, acute coronary syndrome; *CABG*, coronary artery bypass grafting; *CAD*, coronary artery disease; *IQR*, interquartile range; *LCBI*, lipid core burden index; *MI*, myocardial infarction; *PCI*, percutaneous coronary intervention; *SAP*, stable angina pectoris

^1^A significant stenosis was defined as a stenosis ≥50% of the vessel diameter by visual assessment of the coronary angiogram

### Association Between Coronary LCBI and Biomarkers

**Fig. 3:**
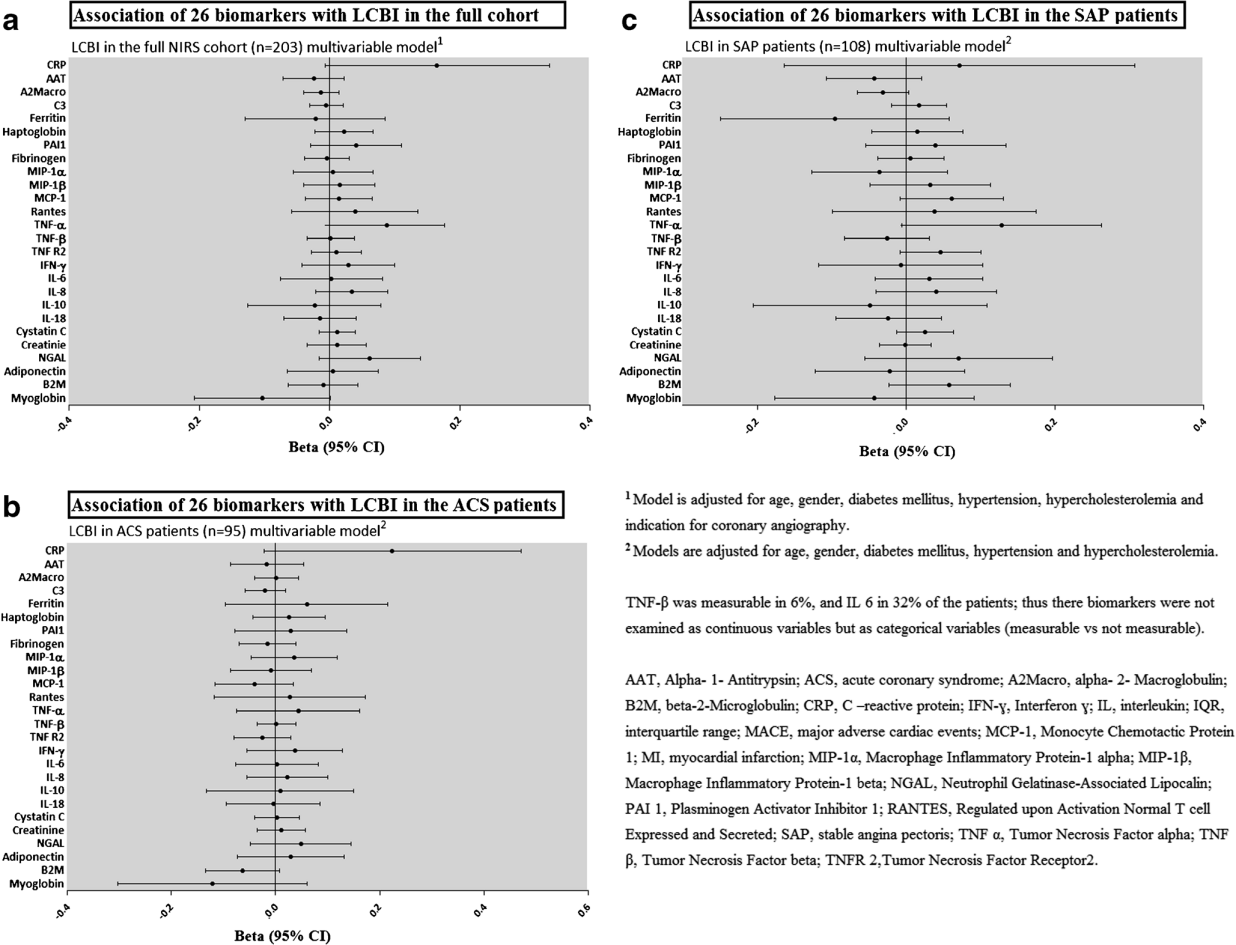
Association of 26 biomarkers with LCBI in the full NIRS cohort and in patients with ACS or SAP. Results are presented as betas which signify unit increase in (Ln-transformed) biomarker concentration or in category of biomarker concentration, per unit increase in Ln-transformed LCBI measurement, with 95% confidence intervals (CI)

The results of the multivariable linear regression analyses are depicted in Fig. 3 and Table 2. Higher TNF-α (multivariable adjusted B 0.088 ln (pg/ml) per unit LCBI; 95% CI 0.000–0.177, *p* = 0.05) displayed a tendency towards an association at the *p* = 0.05 level with higher LCBI in the full cohort after adjustment for clinical characteristics. Effect estimates did not reach statistical significance at the *p* = 0.05 level in ACS and SAP patients (Fig. 3 and Table 2). After Bonferroni correction, no associations were present between any of the biomarkers and LCBI. Results of the univariable analyses were materially the same (results not presented).

**Table 2:** Association between biomarkers and LCBI (NIRS cohort *n* = 203)

	Total (*n* = 203)		ACS patients (*n* = 95)		SAP patients (*n* = 108)	
	B [95% CI]^1^	*p* value	B [95% CI]^2^	*p* value	B [95% CI]^2^	*p* value
Acute phase proteins						
CRP^3^	0.165 (−0.007–0.337)	0.06	0.224 (−0.023–0.471)	0.08	0.071 (−0.164–0.307)	0.55
AAT^4^	−0.025 (−0.071–0.022)	0.30	−0.017 (−0.087–0.054)	0.64	−0.043 (−0.107–0.021)	0.18
A2Macro^5^	−0.013 (−0.040–0.014)	0.35	0.001 (−0.040–0.043)	0.95	−0.031 (−0.065–0.004)	0.08
Complement C3^5^	−0.005 (−0.031–0.021)	0.72	−0.020 (−0.059–0.019)	0.32	0.018 (−0.019–0.054)	0.34
Ferritin^5^	−0.022 (−0.129–0.086)	0.69	0.060 (−0.096–0.215)	0.45	−0.096 (−0.249–0.058)	0.22
Haptoglobin^6^	0.022 (−0.023–0.067)	0.34	0.025 (−0.044–0.095)	0.47	0.015 (−0.046–0.076)	0.62
PAI 1^6^	0.041 (−0.029–0.111)	0.25	0.029 (−0.078–0.136)	0.59	0.040 (−0.054–0.134)	0.40
Fibrinogen^6^	−0.005 (−0.039–0.030)	0.79	−0.015 (−0.070–0.039)	0.58	0.006 (−0.038–0.051)	0.78
Chemokine						
MIP-1 α^6^	0.006 (−0.056–0.067)	0.86	0.036 (−0.047–0.118)	0.39	−0.035 (−0.127–0.056)	0.44
MIP-1 β^6^	0.015 (−0.040–0.070)	0.60	−0.008 (−0.086–0.069)	0.83	0.033 (−0.048–0.113)	0.42
MCP-1^6^	0.015 (−0.037–0.066)	0.57	−0.041 (−0.116–0.034)	0.28	0.062 (−0.008–0.131)	0.08
T cell-specific RANTES^6^	0.039 (−0.058–0.136)	0.43	0.028 (−0.118–0.173)	0.71	0.038 (−0.099–0.175)	0.59
Cytokines						
TNF-α^5^	0.088 (0.000–0.177)	0.05	0.043 (−0.075–0.161)	0.47	0.128 (−0.005–0.262)	0.06
TNF-β^6^	−0.007 (−0.039–0.026)	0.68	0.001 (−0.035–0.038)	0.94	−0.026 (−0.083–0.032)	0.37
TNF R2^6^	0.011 (−0.028–0.049)	0.59	−0.025 (−0.080–0.029)	0.36	0.047 (−0.008–0.101)	0.09
IFN-ɣ^5^	0.029 (−0.042–0.100)	0.42	0.037 (−0.055–0.128)	0.42	−0.007 (−0.117–0.103)	0.90
IL-6^6^	0.018 (−0.034–0.071)	0.49	0.003 (−0.076–0.082)	0.94	0.031 (−0.041–0.103)	0.39
IL-8^6^	0.034 (−0.021–0.090)	0.23	0.022 (−0.056–0.100)	0.57	0.041 (−0.040–0.121)	0.32
IL-10^5^	−0.023 (−0.125–0.079)	0.65	0.009 (−0.132–0.149)	0.90	−0.048 (−0.205–0.109)	0.55
IL-18^5^	−0.015 (−0.070–0.041)	0.61	−0.005 (−0.094–0.085)	0.92	−0.023 (−0.095–0.048)	0.52
Renal markers						
Creatinine^7^	0.004 (−0.024–0.032)	0.77	0.011 (−0.035–0.057)	0.64	−0.001 (−0.035–0.034)	0.96
Cystatin C^6^	0.011 (−0.016–0.039)	0.42	0.002 (−0.040–0.045)	0.91	0.026 (−0.012–0.064)	0.17
NGAL^5^	0.062 (−0.016–0.139)	0.12	0.048 (−0.049–0.145)	0.33	0.070 (−0.055–0.196)	0.27
Other markers						
Adiponectin^6^	0.005 (−0.065–0.075)	0.87	0.028 (−0.074–0.131)	0.59	−0.021 (−0.122–0.079)	0.67
Myoglobin^6^	−0.096 (−0.207–0.015)	0.09	−0.120 (−0.302–0.061)	0.19	−0.042 (−0.176–0.091)	0.53
B2M^5^	−0.010 (−0.063–0.043)	0.72	−0.064 (−0.135–0.007)	0.08	0.059 (−0.023–0.140)	0.15

Variables with a non-normal distribution were transformed by the natural logarithm (ln). Results are presented as beta coefficients (B) that indicate unit increase in (ln-transformed) biomarker level or in category of biomarker concentration, per unit Ln-transformed LCBI, with 95% confidence intervals (CI)

*AAT*, alpha-1-antitrypsin; *ACS*, acute coronary syndrome; *A2Macro*, alpha-2-Macroglobulin; *B2M*, beta-2-microglobulin; *CRP*, C-reactive protein; *IFN-ɣ*, interferon ɣ; IL, interleukin; *IQR*, interquartile range; *LCBI*, lipid core burden index; *MCP-1*, monocyte chemotactic protein 1; *MI*, myocardial infarction; *MIP*-1α, macrophage inflammatory protein-1 alpha; *MIP-1β*, macrophage inflammatory protein-1 beta; *NGAL*, neutrophil gelatinase-associated lipocalin; *PAI* 1, plasminogen activator inhibitor 1; *RANTES*, regulated upon activation normal T cell expressed and secreted; *SAP*, stable angina pectoris; *TNF-*α, tumor necrosis factor alpha; *TNF-β*, tumor necrosis factor beta; *TNF R2*,tumor necrosis factor receptor 2

^1^Model is adjusted for age, gender, diabetes mellitus, hypertension, hypercholesterolemia, and indication for coronary angiography

^2^Models are adjusted for age, gender, diabetes mellitus, hypertension, and hypercholesterolemia

^3^Available in the full cohort

^4^Available in 156 patients

^5^Available in 190 patients

^6^Too low to detect in a large part of the patients (TNF-β was measurable in 6% and IL 6 in 32% of the patients) and thus not examined as a continuous variable but as a categorical variable (measurable vs not measurable)

^7^Available in 188 patients

### Biomarkers and Major Adverse Cardiac Events

**Fig. 4:**
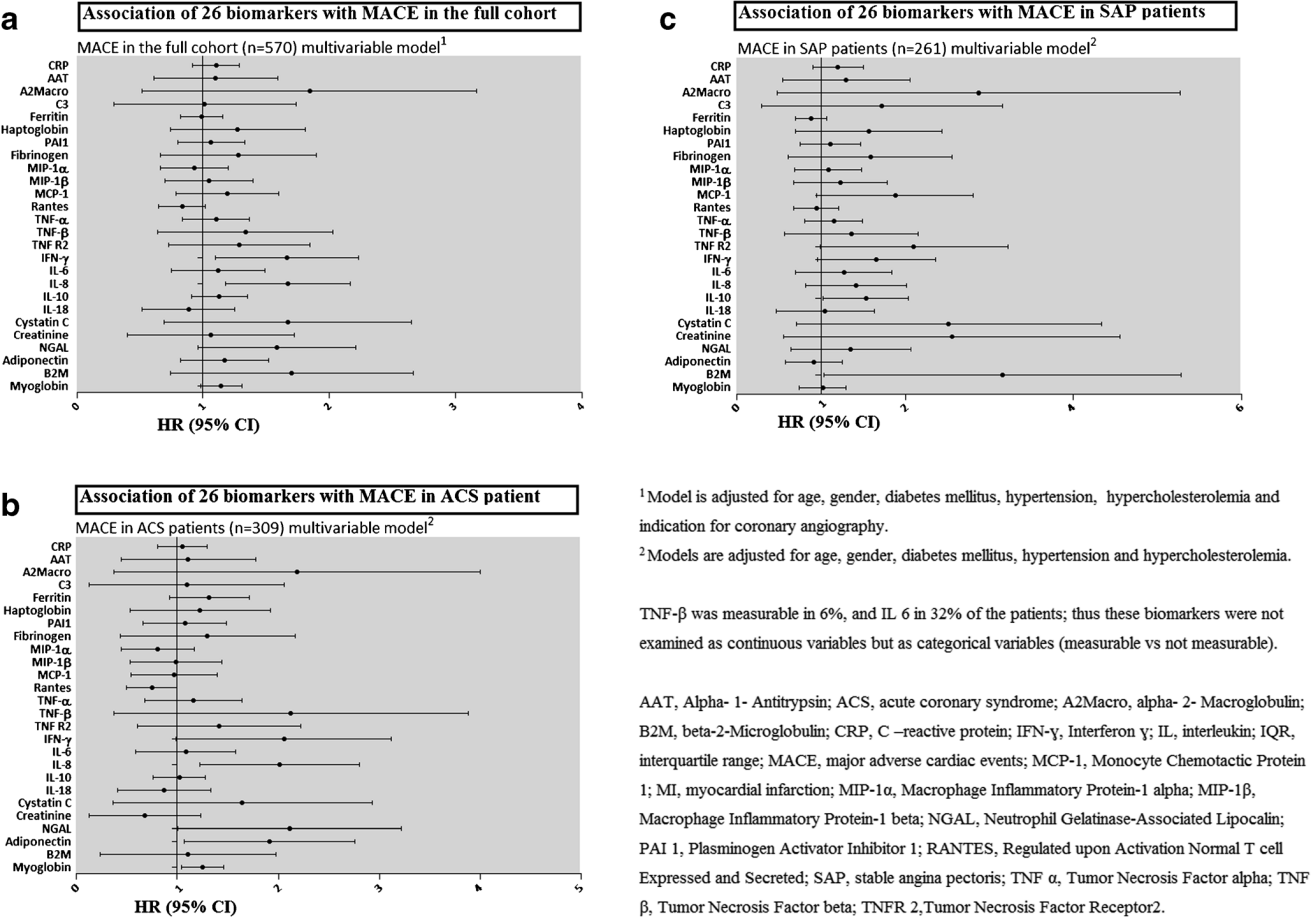
Association of 26 biomarkers with MACE in the full cohort and in patients with ACS or SAP. Results are presented as hazard ratios (HRs) per unit increase in (Ln-transformed) biomarker concentration or per category of biomarker concentration, with 95% confidence intervals (CI)

Vital status was acquired for 569 out of 570 patients (99.8%). The follow-up questionnaire assessing the occurrence of MACE was completed by 87.5% of the 570 patients. During a median follow-up time of 4.7 years IQR: [4.2–5.6] years, 155 patients (27%) experienced at least 1 MACE (primary endpoint). Hazard ratios for the occurrence of MACE are shown in Fig. 4 and Supplemental Tables 4a and 4b. After Bonferroni correction, only IL-8 (*p* = 0.002) was borderline significantly associated with MACE. No independent associations were present between the other biomarkers and MACE. At the *p* = 0.05 level, some biomarkers tended to display associations with MACE. Specifically, on univariable analysis, higher T cell-specific RANTES, IFN-ɣ, IL-8, cystatin C, and B2M were associated with a higher incidence of MACE at the *p* = 0.05 level. After adjustment for clinical characteristics, IFN-ɣ (HR 1.57; 95% CI [1.10–2.23 per ln (pg/ml)) and IL-8 (HR 1.60; 95% CI [1.18–2.17 per ln (pg/ml)) remained independently associated with MACE at the *p* = 0.05 level. Interaction terms between biomarkers and indication for angiography only reached significance for IL-10 (*p* for interaction = 0.05). In patients diagnosed with ACS or SAP, there were no significant associations between any of the biomarkers and MACE.

Only IL-8 (*p* = 0.0015) was significantly associated with the composite of death or ACS (secondary endpoint) after Bonferroni correction and adjustment for clinical characteristics (Supplemental Table 5). At the *p* = 0.05 level, higher CRP, A2Macro, fibrinogen, TNFR2, INF-γ, IL-8, cystatin C, adiponectin, and B2M were associated with this secondary endpoint on univariable analysis (data not shown). Interaction terms between biomarkers and indication for angiography reached significance for B2M (*p* for interaction = 0.036). In patients with ACS, only IL-8 (HR 2.89; 95%CI [1.86–4.14 per ln (pg/ml) *p* ≤ 0.001) remained associated with the composite of death or ACS after multivariable adjustment and Bonferroni correction. In patients with SAP, the HR for IL-8 was closer to the null (HR 1.12; 95% CI [0.61–2.03 per ln (pg/ml) *p* = 0.72). Effect estimates for CRP and INF-γ were similar to those in the full cohort in ACS and SAP patients, but statistical significance was not reached. For B2M, the multivariable adjusted hazard ratio was significantly higher in SAP patients than in ACS patients, but did not reach statistical significance after Bonferroni correction.

## Discussion

We investigated the association of 26 circulating biomarkers with NIRS-derived LCBI in 203 patients undergoing coronary angiography. After multivariable adjustment and correction for multiple testing, none of the 26 biomarkers was associated with LCBI. Furthermore, we also investigated the long-term prognostic value of these 26 biomarkers for clinical cardiovascular outcome in 570 patients. After correction for multiple testing, we found that IL-8 was borderline significantly associated with MACE and independently associated with death or ACS.

Studies on the association between circulating biomarkers and NIRS-derived characteristics have not been performed previously. NIRS has recently progressed from bench testing to human studies. In 1993, Cassis and Lodder [18] first described the use of NIRS for characterization of atherosclerotic plaque in rabbit aortas. Ever since, there have been several [4, 15] studies that have validated the use of NIRS for identification of lipid deposition within the coronary arteries. NIRS has been shown to identify extensive LCPs that are associated with a high risk of peri-procedural myocardial infarction [15]. In our previous report on the current study population, NIRS-derived LCBI was an independent predictor of MACE during 1 year follow-up [6••]. As NIRS has the potential to identify LCPs indicative of plaque vulnerability in the coronary arteries, we hypothesized that circulating inflammatory biomarkers are associated with NIRS-derived LCBI. However, we could not demonstrate any associations between these biomarkers and NIRS-derived LCBI after Bonferroni correction. These results suggest that any potential effects of these biomarkers on atherosclerosis are exerted through other mechanisms.

TNF-α is a pro-inflammatory cytokine with pleiotropic actions. In a previous report on the current patient population, TNF-α concentration was positively associated with plaque burden and plaque vulnerability as determined by IVUS [8]. In the current study, we found that at the *p* = 0.05 level, TNF-α displayed a tendency towards a positive association with LCBI after multivariable adjustment, but this association did not persist after correction for multiple testing. Previously, Sukhija et al. [19] found no association between serum TNF-α levels and extent of atherosclerosis or clinical outcome in patients with known coronary artery disease (CAD). Conversely, other studies [20, 21] have demonstrated positive associations between plasma concentration of TNF-α and coronary events. However, in our study, TNF-α was not associated with MACE at long-term follow-up, implying that the deleterious effect of TNF-α, if any, does not translate into a higher MACE rate in the current study population. More research is necessary to further substantiate the pathological mechanisms underlying the roles of this biomarker in atherosclerotic plaque development. No associations could be demonstrated between any of the other biomarkers and NIRS-derived LCBI.

In our previous reports on the ATHEROREMO-IVUS study [8–12], levels of CRP, ferritin, RANTES, TNF-α, IL-10, cystatin C, and NGAL were associated with IVUS-VH-derived plaque burden in the full cohort. Additionally, IL-10, cystatin C, and NGAL were associated with IVUS-VH-derived thin-cap fibroatheroma (VH-TCFA) lesions. This difference in findings could in part be explained by the differences in definitions used for NIRS- and IVUS-derived measures of plaque vulnerability. NIRS-derived LCBI represents the amount of LCP in the entire scanned artery segment, and is computed as the fraction of valid pixels on the block chemogram that exceed an LCP probability of 0.6, multiplied with 1000. IVUS-VH-derived TCFA lesions are defined as lesions with presence of > 10% confluent necrotic core in direct contact with the lumen [9]. Although these entities are related, previous studies have shown that the correlation between LCP as detected by NIRS and necrotic core as detected by IVUS-VH is weak [14].

We found that IL-8 is associated with clinical outcome during long-term follow-up. Inoue T. et al. [22] investigated the long-term prognostic value of IL-8 in patients with CAD and found IL-8 as only cytokine predictor of cardiovascular events, independently of other cytokines and hs-CRP. Cavusoglu et al. [23] found an association between high baseline plasma levels of IL-8 with increased risk of long-term all-cause mortality in patients with ACS. Our findings are in line with these results.

### Study Limitations

This study has several limitations. First, this is a cross-sectional study. As we did not repeat NIRS imaging of the same segment at a later time point, no information is available on the change in LCBI and its relation with biomarkers levels over time. Future research might focus on the effects of changes in biomarker level and their effect on LCBI. Secondly, the NIRS image acquisition was performed in only one non-culprit vessel. This study design was chosen based on the hypothesis that such a non-stenotic segment reflects coronary wall pathophysiology in the larger coronary tree [13•]. This assumption, on its part, was based on the fact that ex vivo, as well as in vivo, studies in patients with myocardial infarction, have demonstrated presence of TCFA (as assessed by IVUS) located elsewhere than the culprit lesion or even culprit artery [8–12]. In fact, we were subsequently able to confirm this hypothesis, by demonstrating that NIRS imaging characteristics of the non-culprit artery are associated with increased risk of MACE [6••].

## Conclusion

None of the 26 biomarkers we examined was associated with LCBI after correction for multiple testing. IL-8 was associated with clinical outcome in patients undergoing coronary angiography after correction for multiple testing. Altogether, the multiplex panel we investigated here did not render a useful blood biomarker of high LCBI.

## Electronic supplementary material

ESM 1 (DOCX 71 kb)
