# Supplementary material for: Associations of 26 Circulating Inflammatory and Renal Biomarkers with Near-Infrared Spectroscopy and Long-term Cardiovascular Outcome in Patients Undergoing Coronary Angiography (ATHEROREMO-NIRS Substudy)
Source: Curr Atheroscler Rep. 2018 Sep 14;20(10):52. doi: 10.1007/s11883-018-0752-8 (PMC6153584; doi:10.1007/s11883-018-0752-8)
Supplement: Supplementary file 1 — (DOCX 71 kb) [file 11883_2018_752_MOESM1_ESM.docx]

**Supplement**

**Supplemental Table 1. Baseline clinical and procedural characteristics (full cohort, n=570)**

|  | Total  (n=570) | ACS patients  (n=309) | SAP patients  (n= 261) |
| --- | --- | --- | --- |
| Clinical characteristics |  |  |  |
| Age, years, mean ± standard deviation | 61.5 ± 11.4 | 59.7 ± 11.9 | 63.6 ± 10.3 |
| Male, n (%) | 430 (75.4%) | 227 (73.5) | 203 (77.8) |
| Diabetes Mellitus, n (%) | 99 (17.4) | 40 (12.9) | 59 (22.6) |
| Hypertension, n (%) | 295 (51.8) | 134 (43.4) | 161 (61.7) |
| Hypercholesterolemia, n (%) | 317 (55.6) | 137 (44.3) | 180 (69.0) |
| Smoking, n (%) | 164 (28.8) | 115 (37.2) | 49 (18.8) |
| Positive family history of CAD, n (%) | 293 (51.5) | 140 (45.5) | 153 (58.6) |
| Previous MI, n (%) | 184 (32.3) | 80 (25.9) | 104 (39.8) |
| Previous PCI, n (%) | 185 (32.5) | 57 (18.4) | 128 (49.0) |
| Previous CABG, n (%) | 18 (3.2) | 7 (2.3) | 11 (4.2) |
| Previous stroke, n (%) | 23 (4.0) | 10 (3.2) | 13 (5.0) |
| Peripheral artery disease, n (%) | 36 (6.3) | 12 (3.9) | 24 (9.2) |
| History of heart failure, n (%) | 19 (3.3) | 6 (1.9) | 13 (5.0) |
| Procedural characteristics |  |  |  |
| *Indication for coronary angiography* |  |  |  |
| ACS, n (%) | 309 (54.2) | 309 (100) | 0 (0) |
| Acute MI, n (%) | 159 (27.9) | 159 (51.5) | 0 (0) |
| Unstable angina pectoris, n (%) | 150 (26.3) | 150 (48.5) | 0 (0) |
| Stable angina pectoris, n (%) | 261 (45.8) | 0 (0) | 261 (100) |
| PCI performed, n (%) | 501 (87.9) | 287 (92.9) | 214 (82.0) |
| *Coronary artery disease*^[[1]](#footnote-1)^ |  |  |  |
| No significant stenosis, n (%) | 42 (7.4) | 18 (5.8) | 24 (9.2) |
| 1-vessel disease, n (%) | 301 (52.8) | 168 (54.4) | 133 (51.0) |
| 2-vessel disease, n (%) | 166 (29.1) | 88 (28.5) | 78 (29.9) |
| 3-vessel disease, n (%) | 61 (10.7) | 35 11.3) | 26 (10.0) |
| NIRS characteristics |  |  |  |
| *Median LCBI (IQR)* | 43.0 (15.0-84.0) | 47.5 (16.0-90.5) | 35.0 (14.0-80.8) |
| *Imaged coronary artery* |  |  |  |
| Left anterior descending, n (%) | 204 (35.9) | 117 (37.9) | 87 (33.6) |
| Left circumflex, n (%) | 190 (33.5) | 107 (34.6) | 83 (32.0) |
| Right coronary artery, n (%) | 174 (30.6) | 85 (27.5) | 89 (34.4) |
|  |  |  |  |

Continuous variables are presented as mean ± standard deviation (SD) or median [IQR]. Categorical variables are presented in numbers (n) and percentages (%).

ACS, acute coronary syndrome; CABG, coronary artery bypass grafting; CAD, coronary artery disease; IQR, interquartile range; LCBI, Lipid Core Burden Index; MI, myocardial infarction; PCI, percutaneous coronary intervention; SAP, stable angina pectoris.

**Supplemental Table 2. Biomarker concentrations (ATHEROREMO-NIRS cohort, n=203)**

|  | Total  (n=203) | ACS patients  (n=95) | SAP patients  (n=108) |
| --- | --- | --- | --- |
| Acute phase proteins |  |  |  |
| CRP (mg/l)^[[2]](#footnote-2)^ | 2.10[0.80-5.35] | 3.00[1.00-6.65] | 1.60[0.60-4.00] |
| AAT (mg/ml)^[[3]](#footnote-3)^ | 1.50[1.20-1.80] | 1.60[1.20-1.90] | 1.40[1.20-1.65] |
| A2Macro (mg/ml)2 | 1.60[1.40-1.80] | 1.60[1.40-1.80] | 1.60[1.50-1.80] |
| Complement C3 (mg/ml)2 | 0.94[0.78-1.10] | 0.90[0.78-1.10] | 0.96[0.80-1.10] |
| Ferritin (mg/ml)^[[4]](#footnote-4)^ | 178.50[94.0-280.75] | 191.5[98.75-356.0] | 150.50[83.50-237.75] |
| Haptoglobin (mg/ml)3 | 1.43[0.87-2.19] | 1.60[0.96-2.50] | 1.30[0.87-2.10] |
| PAI 1 (ng/ml)3 | 31.0[22.0-45.25] | 31.0[23.0-44.15] | 30.50[20.40-46.75] |
| Fibrinogen (pg/ml)3 | 3.90[3.00-4.70] | 4.05[3.0-5.11] | 3.76[2.93-4.41] |
| Chemokine |  |  |  |
| MIP-1α (pg/ml)3 | 16.0[12.0-23.0] | 15.0[11.0-21.90] | 17.50[12.0-24.50] |
| MIP-1β (pg/ml)3 | 119.50[92.0-154.25] | 126.0[96.0-165.50] | 112.50[90.18-142.0] |
| MCP-1 (pg/ml)3 | 86.5[67.0-108.0] | 85.0[63.75-106.25] | 87.50 [71.25-113.25] |
| T-Cell-Specific RANTES (ng/ml)3 | 9.43[4.98-15.0] | 10.50[5.83-17.25] | 9.34[4.70-14.0] |
| Cytokines |  |  |  |
| TNF-α (pg/ml)2 | 2.00[1.40-2.80] | 1.80[1.40-2.60] | 2.0[1.40-3.30] |
| TNF-β (pg/ml)^[[5]](#footnote-5)^ | 36.0[18.0-119.0] | 37.0[18.0-… ] | 35.0[18.0-128.0] |
| TNF R2 (ng/ml)^6^ | 4.40[3.60-5.60] | 4.40[3.60-6.00] | 4.35[3.53-5.52] |
| IFN-ɣ (pg/ml)2 | 5.40[3.90-7.90] | 5.10[3.90-7.30] | 5.60[3.90-8.50] |
| IL-6 (pg/ml)^[[6]](#footnote-6)^ | 3.10[2.13-5.45] | 9.40[7.85-13.0] | 2.20[2.13-3.84] |
| IL-8 (pg/ml)3 | 9.00[6.90-12.0] | 3.57[2.43-6.90] | 8.34[6.15-10.83] |
| IL-10 (pg/ml)2 | 5.00[3.60-7.25] | 5.70[3.70-8.0] | 4.80[3.20-6.80] |
| IL-18 (pg/ml)2 | 155.0[122.25-190.50] | 150.0[108.0-191.0] | 156.0[131.0-189.0 ] |
| Renal markers |  |  |  |
| Cystatin C (ng/ml)3 | 792.50[687.75-926.5] | 788.5[665.5-931.5] | 806.0[711.25-921.75] |
| Creatinine (umol/l)^[[7]](#footnote-7)^ | 75.00[62.00-84.50] | 72.50[61.00-84.50] | 76.00[62.50-84.50] |
| NGAL (pg/ml)^[[8]](#footnote-8)^ | 194.50[137.0-239.0] | 201.0[143.0-254.0] | 175.0[125.0-238.5] |
| Other markers |  |  |  |
| Adiponectin (pg/ml)3 | 2.90[1.80-4.10] | 2.75[1.80-4.03] | 3.00[1.90-4.18] |
| B2M (pg/ml)2 | 1.35[1.10-1.70] | 1.30[1.0-1.70] | 1.40[1.20-1.80] |
| Myoglobin (pg/ml)3 | 32.50[19.75-60.50] | 33.50[21.0- 69.25] | 31.0[18.0-55.58] |

All biomarkers are presented as median [IQR].

AAT, Alpha- 1- Antitrypsin; ACS, acute coronary syndrome; A2Macro, alpha- 2- Macroglobulin; B2M, beta-2-Microglobulin; CRP, C –reactive protein; IFN-ɣ, Interferon ɣ; IL, interleukin; IQR, interquartile range; MCP-1, Monocyte Chemotactic Protein 1; MI, myocardial infarction; MIP-1α, Macrophage Inflammatory Protein-1 alpha; MIP-1β, Macrophage Inflammatory Protein-1 beta; NGAL, Neutrophil Gelatinase-Associated Lipocalin; PAI 1, Plasminogen Activator Inhibitor 1; RANTES, Regulated upon Activation Normal T cell Expressed and Secreted; SAP, stable angina pectoris; TNF-α, Tumor Necrosis Factor alpha; TNF-β, Tumor Necrosis Factor beta; TNF R2,Tumor Necrosis Factor Receptor2.

**Supplemental Table 3. Biomarker concentrations in the full cohort (n=570)**

|  | Total  (n=570) | ACS patients  (n=309) | SAP patients  (n= 261) |
| --- | --- | --- | --- |
| Acute phase proteins |  |  |  |
| CRP (mg/l), median [IQR]^[[9]](#footnote-9)^^,^^[[10]](#footnote-10)^ | 2.10 [0.83-5.28] | 2.80 [1.10-6.95] | 1.45 [0.60-3.05] |
| AAT (mg/ml), median [IQR]^[[11]](#footnote-11)^ | 1.40 [1.20-1.70] | 1.40 [1.20-1.70] | 1.40 [1.20-1.65] |
| A2Macro (mg/ml),median [IQR]^3^ | 1.50 [1.40-1.80] | 1.50 [1.30-1.80] | 1.60 [1.40-1.80] |
| Complement C3 (mg/ml), median [IQR]^3^ | 0.90 [0.78-1.10] | 0.90 [0.78-1.10] | 0.92 [0.79-1.00] |
| Ferritin (mg/ml), median [IQR]^1^ | 173.50 [94.75-283.0] | 191.0 [102.50-320.50] | 144.0 [82.0-242.50] |
| Haptoglobin (mg/ml), median [IQR]^1,2^ | 1.40 [0.93-2.10] | 1.50 [0.99-2.20] | 1.30 [0.86-1.90] |
| PAI 1(ng/ml), median [IQR]^1^ | 35.0 [23.0-53.0] | 38.0 [27.0-61.50] | 31.0 [21.0-46.0] |
| Fibrinogen (pg/ml), median [IQR]^1^ | 3.50 [2.90-4.40] | 3.60 [3.0-4.53] | 3.40 [2.80-4.30] |
| Chemokine |  |  |  |
| MIP-1 α (pg/ml), median [IQR]^[[12]](#footnote-12)^ | 16.0 [12.0-21.90] | 15.0 [12.0-21.90] | 17.0 [12.0-21.92] |
| MIP-1 β (pg/ml), median [IQR]^1,2^ | 123.0 [92.0-165.0] | 129.50 [95.0-177.25] | 114.0 [89.45-146.0] |
| MCP-1 (pg/ml), median [IQR]^1,2^ | 91.0 [70.0-121.75] | 92.0 [70.0-133.0] | 88.0 [70.50-111.0] |
| T-Cell-Specific RANTES (ng/ml), median [IQR]^1^ | 11.0 [6.38-19.0] | 14.0 [7.55-23.0] | 9.06 [5.0-14.25] |
| Cytokines |  |  |  |
| TNF-α (pg/ml), median [IQR]^[[13]](#footnote-13)^ | 1.95 [1.40-2.90] | 1.80 [1.40-2.60] | 2.0 [1.40-3.25] |
| TNF-β (pg/ml), median [IQR]^[[14]](#footnote-14)^ | 35.0 [18.0-116.0] | 20.50 [16.50-44.25] | 36.5 [27.0-152.75] |
| TNF R2 (ng/ml), median [IQR]^1,2^ | 4.50 [3.60-5.71] | 4.40 [3.50-5.80] | 4.50 [3.65-5.64] |
| IFN-ɣ (pg/ml), median [IQR]^3^ | 5.10 [3.85-7.30] | 4.80 [3.80-6.60] | 5.70 [4.20-8.23] |
| IL-6 (pg/ml), median [IQR]^[[15]](#footnote-15)^ | 3.50 [2.20-5.90] | 3.70 [2.50-6.79] | 2.50 [2.13-4.07] |
| IL-8 (pg/ml), median [IQR]^1,2^ | 9.00 [6.80-12.0] | 9.90 [7.30-13.0] | 8.34 [6.53-10.83] |
| IL-10 (pg/ml), median [IQR]^3^ | 5.20 [3.63-9.40] | 6.90 [4.10-15.0] | 4.50 [3.0-6.0] |
| IL-18 (pg/ml), median [IQR]^3^ | 171.0 [132.50-215.0] | 173.0 [134.0-217.0] | 169.50 [130.50-211.25] |
| Renal markers |  |  |  |
| Creatinine (umol/l), median [IQR]^1,2^ | 77 [66.0-86.50] | 77.0 [65.0-87.0] | 76.0 [67.0-86.0] |
| Cystatin C (mg/ml), median [IQR]^1^ | 796.0 [691.0-923.0] | 791.0 [674.5-915.5] | 802.0 [712.50-935.50] |
| NGAL (pg/ml), median [IQR]^[[16]](#footnote-16)^ | 201.0 [148.0-260.0] | 207.0 [149.0-282.50] | 186.0 [143.0-242.75] |
| Other markers |  |  |  |
| Adiponectin, median [IQR]^1^ | 2.80 [1.90-4.00] | 2.80 [1.88-4.10] | 2.86 [1.90-3.90] |
| Myoglobin (pg/ml), median [IQR]^1,2^ | 38.0 [22.0-79.0] | 51.0 [23.0- 190.0] | 31.0 [20.0-50.50] |
| B2M (pg/ml), median [IQR]^3^ | 1.30 [1.10-1.70] | 1.30 [1.0-1.70] | 1.40 [1.20-1.70] |

All biomarkers are presented as median [IQR].

AAT, Alpha-1-Antitrypsin; ACS, acute coronary syndrome; A2Macro, alpha-2-Macroglobulin; B2M, beta-2-Microglobulin; CRP, C–reactive protein; IFN-ɣ, Interferon ɣ; IL, interleukin; IQR, interquartile range; MCP-1, Monocyte Chemotactic Protein 1; MI, myocardial infarction; MIP-1α, Macrophage Inflammatory Protein-1 alpha; MIP-1β, Macrophage Inflammatory Protein-1 beta; NGAL, Neutrophil Gelatinase-Associated Lipocalin; PAI 1, Plasminogen Activator Inhibitor 1; RANTES, Regulated upon Activation Normal T cell Expressed and Secreted; SAP, stable angina pectoris; TNF-α, Tumor Necrosis Factor alpha; TNF-β, Tumor Necrosis Factor beta; TNF R2,Tumor Necrosis Factor Receptor2.

**Supplemental Table 4a. Univariate association between biomarkers with major adverse cardiac events (MACE; composite of all-cause mortality, nonfatal ACS or unplanned coronary revascularization) in the full cohort (n=570)**

|  | Total (n=570) | | ACS patients (n=309) | | SAP patients (n=261) | |
| --- | --- | --- | --- | --- | --- | --- |
|  | HR [95% CI] | P-value | HR [95% CI] | P-value | HR [95% CI] | P-value |
| Acute phase proteins |  |  |  |  |  |  |
| CRP^[[17]](#footnote-17)^ | 1.05 (0.89-1.24) | 0.56 | 1.10 (0.88-1.38) | 0.41 | 1.18 (0.92-1.50) | 0.19 |
| AAT^[[18]](#footnote-18)^ | 1.00 (0.63-1.58) | 0.99 | 1.03 (0.53-2.00) | 0.93 | 1.06 (0.56-2.02) | 0.86 |
| A2Macro^2^ | 1.91 (0.87-4.24) | 0.11 | 1.53 (0.47-4.95) | 0.48 | 1.84 (0.58-5.84) | 0.30 |
| Complement C3^2^ | 0.74 (0.31-1.78) | 0.51 | 0.56 (0.15-2.14) | 0.40 | 0.98 (031-3.13) | 0.98 |
| Ferritin^1^ | 0.94 (0.79-1.12) | 0.50 | 1.20 (0.88-1.62) | 0.25 | 0.89 (0.72-1.09) | 0.26 |
| Haptoglobin^1^ | 1.11 (0.72-1.69) | 0.64 | 1.16 (0.62-2.16) | 0.64 | 1.34 (0.74-2.44) | 0.34 |
| PAI 1^1^ | 0.91 (0.70-1.18) | 0.47 | 0.91 (0.61-1.35) | 0.62 | 1.08 (0.77-1.52) | 0.66 |
| Fibrinogen^1^ | 1.23 (0.75-2.01) | 0.42 | 1.30 (0.63-2.68) | 0.48 | 1.35 (0.68-2.69) | 0.39 |
| Chemokine |  |  |  |  |  |  |
| MIP-1 α^1^ | 0.96 (0.72-1.28) | 0.78 | 0.85 (0.54-1.34) | 0.49 | 1.01 (0.69-1.47) | 0.96 |
| MIP-1 β^1^ | 0.93 (0.67-1.30) | 0.66 | 0.94 (0.58-1.54) | 0.82 | 1.09 (0.68-1.75) | 0.73 |
| MCP-1^1^ | 1.09 (0.79-1.51 ) | 0.61 | 0.92 (0.59-1.44) | 0.72 | 1.75 (1.03-2.96) | 0.04 |
| T-Cell-Specific RANTES^1^ | 0.72 (0.58-0.90) | 0.003 | 0.65 (0.47-0.92) | 0.01 | 0.90 (0.67-1.21) | 0.49 |
| Cytokines |  |  |  |  |  |  |
| TNF α^2^^,^^[[19]](#footnote-19)^ | 1.16 (0.91-1.48) | 0.23 | 1.09 (0.71-1.68) | 0.70 | 1.14 (0.85-1.52) | 0.39 |
| TNF β^[[20]](#footnote-20)^ | 1.31 (0.74-2.30) | 0.36 | 1.22 (0.38-3.90) | 0.73 | 1.12 (0.58-2.16) | 0.74 |
| TNFR 2^1^ | 1.47 (0.96-2.27) | 0.08 | 1.16 (0.61-2.22) | 0.65 | 1.78 (0.99-3.22) | 0.06 |
| IFN-ɣ^2^ | 1.79 (1.28- 2.51) | 0.001 | 1.88 (1.09-3.25) | 0.02 | 1.56(1.00- 2.44) | 0.05 |
| IL-6^4^ | 0.93(0.67-1.29) | 0.67 | 0.99 (0.61-1.61) | 0.97 | 1.15 (0.72-1.84) | 0.56 |
| IL-8^1^ | 1.63 (1.21-2.20) | 0.001 | 2.17 (1.47-3.20) | <0.001 | 1.39 (0.90-2.15) | 0.14 |
| IL-10^2^ | 1.00 (0.83-1.20) | 0.98 | 0.99 (0.77-1.27) | 0.93 | 1.45 (1.03-2.05) | 0.03 |
| IL-18^2^ | 0.82 (0.54-1.24) | 0.35 | 0.74 (0.41-1.34) | 0.32 | 0.97 (0.52-1.80) | 0.92 |
| Renal markers |  |  |  |  |  |  |
| Creatinine^1,^^[[21]](#footnote-21)^ | 1.11 (0.58-2.13) | 0.76 | 0.67 (0.24-1.85) | 0.44 | 1.81 (0.74-4.41) | 0.19 |
| Cystatin C^1^ | 1.91 (1.04-3.53) | 0.04 | 1.97 (0.79-4.90) | 0.15 | 1.78 (0.77-4.13) | 0.18 |
| NGAL^2^ | 1.37 (0.91-2.05) | 0.13 | 1.94 (1.01-3.51) | 0.03 | 1.16 (0.65-2.06) | 0.61 |
| Other markers |  |  |  |  |  |  |
| Adiponectin^1^ | 1.09 (0.83-1.43) | 0.54 | 1.79(1.18-2.72) | 0.01 | 0.78 (0.56-1.10) | 0.16 |
| Myoglobin^1^ | 1.04 (0.92-1.18) | 0.53 | 1.20 (1.02-1.40) | 0.03 | 1.05 (0.78-1.31) | 0.91 |
| B2M^2^ | 1.81 (1.02-3.21) | 0.04 | 1.25 (0.51-3.10) | 0.63 | 2.17 (1.05-4.52) | 0.04 |

Results are presented as hazard ratios (HRs) per unit increase in (Ln-transformed) biomarker concentration or per category of biomarker concentration, with 95 % confidence intervals (CI).

AAT, Alpha- 1- Antitrypsin; ACS, acute coronary syndrome; A2Macro, alpha- 2- Macroglobulin; B2M, beta-2-Microglobulin; CRP, C –reactive protein; IFN-ɣ, Interferon ɣ; IL, interleukin; IQR, interquartile range; MACE, major adverse cardiac events; MCP-1, Monocyte Chemotactic Protein 1; MI, myocardial infarction; MIP-1α, Macrophage Inflammatory Protein-1 alpha; MIP-1β, Macrophage Inflammatory Protein-1 beta; NGAL, Neutrophil Gelatinase-Associated Lipocalin; PAI 1, Plasminogen Activator Inhibitor 1; RANTES, Regulated upon Activation Normal T cell Expressed and Secreted; SAP, stable angina pectoris; TNF α, Tumor Necrosis Factor alpha; TNF β, Tumor Necrosis Factor beta; TNFR 2,Tumor Necrosis Factor Receptor2.

**Supplemental Table 4b. Multivariable adjusted association between biomarkers with major adverse cardiac events (MACE; composite of all-cause mortality, nonfatal ACS or unplanned coronary revascularization) in the full cohort (n=570)**

|  | Total (n=570) | | ACS patients (n=309) | | SAP patients (n=261) | |
| --- | --- | --- | --- | --- | --- | --- |
|  | HR [95% CI]^[[22]](#footnote-22)^ | P-value | HR [95% CI]^[[23]](#footnote-23)^ | P-value | HR [95% CI]^2^ | P-value |
| Acute phase proteins |  |  |  |  |  |  |
| CRP^[[24]](#footnote-24)^ | 1.09 (0.92-1.29) | 0.35 | 1.03 (0.81-1.30) | 0.82 | 1.16 (0.90-1.50) | 0.24 |
| AAT^[[25]](#footnote-25)^ | 0.99 (0.61-1.59) | 0.95 | 0.88 (0.44-1.78) | 0.73 | 1.05 (0.54-2.06) | 0.88 |
| A2Macro^4^ | 1.37 (0.59-3.17) | 0.47 | 1.21 (0.37-4.00) | 0.75 | 1.58 (0.48-5.27) | 0.45 |
| Complement C3^4^ | 0.72 (0.29-1.74) | 0.46 | 0.52 (0.13-2.06) | 0.35 | 0.96 (0.29-3.16) | 0.94 |
| Ferritin^3^ | 0.97 (0.82-1.16) | 0.77 | 1.25 (0.92-1.71) | 0.15 | 0.87 (0.70-1.07) | 0.18 |
| Haptoglobin^3^ | 1.16 (0.74-1.81) | 0.52 | 1.01 (0.53-1.92) | 0.98 | 1.31 (0.70-2.44) | 0.40 |
| PAI 1^3^ | 1.03 (0.80-1.33) | 0.83 | 0.99 (0.66-1.49) | 0.96 | 1.05 (0.75-1.47) | 0.77 |
| Fibrinogen^3^ | 1.12 (0.66-1.90) | 0.67 | 0.96 (0.43-2.17) | 0.96 | 1.25 (0.61-2.56) | 0.55 |
| Chemokine |  |  |  |  |  |  |
| MIP-1 α^3,^^[[26]](#footnote-26)^ | 0.89 (0.66-1.20) | 0.44 | 0.71 (0.44-1.17) | 0.19 | 1.01 (0.69-1.48) | 0.97 |
| MIP-1 β^3^ | 0.99 (0.70-1.40) | 0.96 | 0.87(0.53-1.44) | 0.60 | 1.09 (0.67-1.78) | 0.73 |
| MCP-1^3^ | 1.13 (0.79-1.60) | 0.51 | 0.87 (0.54-1.40) | 0.58 | 1.64 (0.95-2.81) | 0.07 |
| T-Cell-Specific RANTES^3^ | 0.82 (0.65-1.02) | 0.07 | 0.71 (0.50-1.00) | 0.05 | 0.91 (0.68-1.21) | 0.50 |
| Cytokines |  |  |  |  |  |  |
| TNF α^4,^^[[27]](#footnote-27)^ | 1.07 (0.84-1.37) | 0.59 | 1.06(0.68-1.64) | 0.81 | 1.10 (0.81-1.49) | 0.54 |
| TNF β^[[28]](#footnote-28)^ | 1.14 (0.64- 2.03) | 0.66 | 1.20 (0.37-3.88) | 0.76 | 1.11 (0.57-2.16) | 0.76 |
| TNFR 2^3^ | 1.16 (0.73-1.85) | 0.52 | 0.73 (0.36-1.48) | 0.38 | 1.68 (0.91-3.13) | 0.10 |
| IFN-ɣ^4^ | 1.57 (1.10- 2.23) | 0.012 | 1.75 (0.99-3.12) | 0.06 | 1.51 (0.96- 2.36) | 0.08 |
| IL-6^[[29]](#footnote-29)^ | 1.06 (0.75-1.49) | 0.74 | 0.97 (0.59-1.58) | 0.89 | 1.14 (0.70-1.84) | 0.59 |
| IL-8^3^ | 1.60 (1.18-2.17) | 0.002 | 1.85 (1.22-2.80) | 0.004 | 1.30 (0.82-2.01) | 0.26 |
| IL-10^4^ | 1.11 (0.91-1.35) | 0.32 | 0.99 (0.76-1.28) | 0.91 | 1.44 (1.02-2.04) | 0.04 |
| IL-18^4^ | 0.80 (0.52-1.22) | 0.29 | 0.74 (0.41-1.33) | 0.31 | 0.87 (0.47-1.63) | 0.66 |
| Renal markers |  |  |  |  |  |  |
| Creatinine^[[30]](#footnote-30)^ | 0.83 (0.40-1.72) | 0.61 | 0.39 (0.13-1.23) | 0.11 | 1.59 (0.56-4.55) | 0.39 |
| Cystatin C^3^ | 1.36 (0.69-2.65) | 0.38 | 1.03 (0.36-2.93) | 0.96 | 1.75 (0.71-4.33) | 0.23 |
| NGAL^4^ | 1.46 (0.96-2.21) | 0.08 | 1.80 (1.01-3.22) | 0.05 | 1.15 (0.64-2.07) | 0.65 |
| Other markers |  |  |  |  |  |  |
| Adiponectin^3^ | 1.12 (0.82-1.52) | 0.47 | 1.72 (1.07-2.76) | 0.03 | 0.85 (0.58-1.25) | 0.41 |
| Myoglobin^3^ | 1.13 (0.98-1.31) | 0.09 | 1.23 (1.04-1.46) | 0.02 | 0.98 (0.74-1.30) | 0.98 |
| B2M^4^ | 1.41 (0.74-2.67) | 0.30 | 0.68 (0.23-1.98) | 0.48 | 2.33 (1.03-5.28) | 0.04 |

Results are presented as hazard ratios (HRs) per unit increase in (Ln-transformed) biomarker concentration or per category of biomarker concentration, with 95 % confidence intervals (CI).

AAT, Alpha- 1- Antitrypsin; ACS, acute coronary syndrome; A2Macro, alpha- 2- Macroglobulin; B2M, beta-2-Microglobulin; CRP, C –reactive protein; IFN-ɣ, Interferon ɣ; IL, interleukin; IQR, interquartile range; MACE, major adverse cardiac events; MCP-1, Monocyte Chemotactic Protein 1; MI, myocardial infarction; MIP-1α, Macrophage Inflammatory Protein-1 alpha; MIP-1β, Macrophage Inflammatory Protein-1 beta; NGAL, Neutrophil Gelatinase-Associated Lipocalin; PAI 1, Plasminogen Activator Inhibitor 1; RANTES, Regulated upon Activation Normal T cell Expressed and Secreted; SAP, stable angina pectoris; TNF α, Tumor Necrosis Factor alpha; TNF β, Tumor Necrosis Factor beta; TNFR 2,Tumor Necrosis Factor Receptor2.

**Supplemental Table 5. Multivariable adjusted association between biomarkers with the composite of all-cause mortality or nonfatal ACS (secondary endpoint) in the full cohort (n=570)**

|  | Total (n=570) | | ACS patients (n=309) | | SAP patients (n=261) | |
| --- | --- | --- | --- | --- | --- | --- |
|  | HR [95% CI]^[[31]](#footnote-31)^ | P-value | HR [95% CI]^[[32]](#footnote-32)^ | P-value | HR [95% CI]^2^ | P-value |
| Acute phase proteins |  |  |  |  |  |  |
| CRP^[[33]](#footnote-33)^ | 1.27 (1.05-1.54) | 0.02 | 1.24 (0.96-1.58) | 0.10 | 1.31 (0.95-1.79) | 0.10 |
| AAT^[[34]](#footnote-34)^ | 1.49 (0.87-2.55) | 0.14 | 1.22 (0.59-2.52) | 0.60 | 1.95 (0.86-4.45) | 0.11 |
| A2Macro^4^ | 1.93 (0.70-5.35) | 0.21 | 1.83 (0.49-6.84) | 0.37 | 2.26 (0.43-11.78) | 0.33 |
| Complement C3^4^ | 1.34 (0.46-3.89) | 0.59 | 0.87 (0.19-3.92) | 0.86 | 2.20 (0.47-10.23) | 0.32 |
| Ferritin^3^ | 1.04 (0.83-1.30) | 0.76 | 1.47 (1.04-2.09) | 0.03 | 0.80 (0.60-1.07) | 0.13 |
| Haptoglobin^3^ | 1.41 (0.82- 2.43) | 0.21 | 1.23 (0.60-2.53) | 0.58 | 1.63 (0.71- 3.72) | 0.25 |
| PAI 1^3^ | 1.27 (0.93-1.75) | 0.13 | 1.16 (0.73-1.84) | 0.53 | 1.45 (0.93-2.26) | 0.10 |
| Fibrinogen^3^ | 1.75 (0.92-3.31) | 0.09 | 1.15 (0.62-3.67) | 0.36 | 1.98 (0.76-5.16) | 0.16 |
| Chemokine |  |  |  |  |  |  |
| MIP-1 α^3,^^[[35]](#footnote-35)^ | 0.91 (0.63-1.32) | 0.63 | 0.86 (0.50-1.48) | 0.85 | 0.95 (0.57-1.56) | 0.83 |
| MIP-1 β^3^ | 0.87 (0.57-1.35) | 0.54 | 1.08 (0.61-1.88) | 0.80 | 0.64 (0.32-1.26) | 0.20 |
| MCP-1^3^ | 0.91 (0.69-1.57) | 0.66 | 0.87 (0.51-1.50) | 0.62 | 1.00 (0.47-2.09) | 0.99 |
| T-Cell-Specific RANTES^3^ | 0.84 (0.64-1.10) | 0.19 | 0.67 (0.45-0.98) | 0.039 | 1.07 (0.73-1.56) | 0.73 |
| Cytokines |  |  |  |  |  |  |
| TNF α^4,^^[[36]](#footnote-36)^ | 1.00 (0.72-1.38) | 0.99 | 1.17 (0.73-1.88) | 0.53 | 0.88 (0.57-1.37) | 0.58 |
| TNF β^[[37]](#footnote-37)^ | 1.56 (0.80-3.03) | 0.19 | 1.69 (0.52-5.51) | 0.38 | 1.41 (0.62-3.18) | 0.41 |
| TNFR 2^3^ | 1.23 (0.69-2.18) | 0.48 | 1.10 (0.50-2.43) | 0.81 | 1.41 (0.61-3.29) | 0.43 |
| IFN-ɣ^4^ | 1.58 (1.03- 2.43) | 0.04 | 1.68 (0.89-3.18) | 0.11 | 1.54 (0.85-2.78) | 0.16 |
| IL-6^[[38]](#footnote-38)^ | 1.25 (0.83-1.89) | 0.28 | 1.63 (0.93-2.86) | 0.09 | 0.86 (0.44-1.67) | 0.66 |
| IL-8^3^ | 1.75 (1.24-2.48) | 0.0015 | 2.89 (1.86-4.14) | <0.001 | 1.12 (0.61-2.03) | 0.72 |
| IL-10^4^ | 1.14 (0.90-1.45) | 0.27 | 1.10 (0.83-1.46) | 0.52 | 1.35 (0.86-2.12) | 0.20 |
| IL-18^4^ | 0.90 (0.54-1.50) | 0.70 | 0.88 (0.47-1.67) | 0.70 | 0.97 (0.42-2.26) | 0.95 |
| Renal markers |  |  |  |  |  |  |
| Creatinine ^[[39]](#footnote-39)^ | 0.85 (0.35-2.06) | 0.71 | 0.56 (0.16-1.96) | 0.36 | 1.49 (0.38-5.93) | 0.57 |
| Cystatin C^3^ | 1.83 (0.82-4.09) | 0.14 | 2.05 (0.68-6.02) | 0.20 | 1.68 (0.51-5.53) | 0.39 |
| NGAL^4^ | 1.44 (0.87-2.39) | 0.16 | 2.66 (1.37-5.19) | 0.004 | 0.60 (0.28-1.27) | 0.18 |
| Other markers |  |  |  |  |  |  |
| Adiponectin^3^ | 1.18 (0.81-1.73) | 0.39 | 2.03 (1.20-3.44) | 0.008 | 0.69 (0.43-1.11) | 0.12 |
| Myoglobin^3^ | 1.14 (0.96-1.35) | 0.13 | 1.23 (1.01-1.49) | 0.04 | 1.02 (0.70-1.49) | 0.90 |
| B2M^4^ | 2.29 (1.04-5.06) | 0.04 | 1.52 (0.48-4.83) | 0.48 | 3.45 (1.14-10.44) | 0.03 |

Results are presented as hazard ratios (HRs) per unit increase in (Ln-transformed) biomarker concentration or per category of biomarker concentration, with 95 % confidence intervals (CI).

AAT, Alpha- 1- Antitrypsin; ACS, acute coronary syndrome; A2Macro, alpha- 2- Macroglobulin; B2M, beta-2-Microglobulin; CRP, C –reactive protein; IFN-ɣ, Interferon ɣ; IL, interleukin; IQR, interquartile range; MACE, major adverse cardiac events; MCP-1, Monocyte Chemotactic Protein 1; MI, myocardial infarction; MIP-1α, Macrophage Inflammatory Protein-1 alpha; MIP-1β, Macrophage Inflammatory Protein-1 beta; NGAL, Neutrophil Gelatinase-Associated Lipocalin; PAI 1, Plasminogen Activator Inhibitor 1; RANTES, Regulated upon Activation Normal T cell Expressed and Secreted; SAP, stable angina pectoris; TNF α, Tumor Necrosis Factor alpha; TNF β, Tumor Necrosis Factor beta; TNFR 2,Tumor Necrosis Factor Receptor2.

1. A significant stenosis was defined as a stenosis ≥ 50% of the vessel diameter by visual assessment of the coronary angiogram.

   [↑](#footnote-ref-1)
2. Available in 201 patients [↑](#footnote-ref-2)
3. Available in a random subset of 156 patients [↑](#footnote-ref-3)
4. Available in 190 patients [↑](#footnote-ref-4)
5. TNF-β was measurable in 6% of 156 patients, too low to detect in 94%. [↑](#footnote-ref-5)
6. IL-6 was measurable in 32% of 190 patients, too low to detect in 68%. [↑](#footnote-ref-6)
7. Available in 99 % of 190 patients, missing in 1 %. [↑](#footnote-ref-7)
8. NGAL was measurable in 96% of 156 patients, too low to detect in 4%. [↑](#footnote-ref-8)
9. Blood samples available in 570 patients. [↑](#footnote-ref-9)
10. Measurable in 99% of 570 patients, too low to detect in 1%. [↑](#footnote-ref-10)
11. Blood samples available in 473 patients. [↑](#footnote-ref-11)
12. MIP-1 α was measurable in 84% of 570 patients, too low to detect in 16% [↑](#footnote-ref-12)
13. TNF- α was measurable in 92% of 473 patients, too low to detect in 8%. [↑](#footnote-ref-13)
14. TNF-β was measurable in 8% of 473 patients, too low to detect in 92%. [↑](#footnote-ref-14)
15. IL-6 was measurable in 38% of 570 patients, too low to detect in 62%. [↑](#footnote-ref-15)
16. NGAL was measurable in 97% of 473 patients, too low to detect in 3%. [↑](#footnote-ref-16)
17. Available in 570 patients. [↑](#footnote-ref-17)
18. Available in 473 patients. [↑](#footnote-ref-18)
19. Measurable in 92% of 473 patients, too low to detect in 8%. [↑](#footnote-ref-19)
20. Too low to detect in in a large part of the patients (TNF-β was measurable in 8% and IL 6 in 38% of the patients) and thus these biomarkers were not examined as continuous variables but as categorical variables (measurable vs not measurable). [↑](#footnote-ref-20)
21. Measurable in 99 % of 570 patients, too low to detect in 1 %. [↑](#footnote-ref-21)
22. Model is adjusted for age, gender, diabetes mellitus, hypertension, hypercholesterolemia and indication for coronary angiography. [↑](#footnote-ref-22)
23. Models are adjusted for age, gender, diabetes mellitus, hypertension and hypercholesterolemia. [↑](#footnote-ref-23)
24. Available in 570 patients. [↑](#footnote-ref-24)
25. Available in 473 patients. [↑](#footnote-ref-25)
26. Measurable in 84% of 570 patients, too low to detect in 16% [↑](#footnote-ref-26)
27. Measurable in 92% of 473 patients, too low to detect in 8%. [↑](#footnote-ref-27)
28. Too low to detect in a large part of the patients (TNF β measurable in 8% of 473 patients, too low to detect in 92), and thus these biomarkers were not examined as continuous variables but as categorical variables (measurable vs not measurable). [↑](#footnote-ref-28)
29. Too low to detect in a large part of the patients (IL-6 measurable in 38% of 570 patients, too low to detect in 62%), and thus these biomarkers were not examined as continuous variables but as categorical variables (measurable vs not measurable). [↑](#footnote-ref-29)
30. Measurable in 99% of 570 patients, too low to detect in 1%. [↑](#footnote-ref-30)
31. Model is adjusted for age, gender, diabetes mellitus, hypertension, hypercholesterolemia and indication for coronary angiography. [↑](#footnote-ref-31)
32. Models are adjusted for age, gender, diabetes mellitus, hypertension and hypercholesterolemia. [↑](#footnote-ref-32)
33. Available in 570 patients. [↑](#footnote-ref-33)
34. Available in 473 patients. [↑](#footnote-ref-34)
35. Measurable in 84% of 570 patients, too low to detect in 16% [↑](#footnote-ref-35)
36. Measurable in 92% of 473 patients, too low to detect in 8%. [↑](#footnote-ref-36)
37. TNF β measurable in 8% of 473 patients, too low to detect in 92 %; thus these biomarkers were not examined as continuous variables but as categorical variables (measurable vs not measurable). [↑](#footnote-ref-37)
38. IL-6 measurable in 38% of 570 patients, too low to detect in 62 %; thus these biomarkers were not examined as continuous variables but as categorical variables (measurable vs not measurable). [↑](#footnote-ref-38)
39. Measurable in 99% of 570 patients, too low to detect in 1%. [↑](#footnote-ref-39)
